# A Yellow Flower With Jaundice Power: Liver Injury Attributed to Greater Celandine

**DOI:** 10.14309/crj.0000000000001347

**Published:** 2024-04-26

**Authors:** Sydney Power, A. Sidney Barritt

**Affiliations:** 1 Department of Medicine, University of North Carolina, Chapel Hill, NC; 2 UNC Liver Center, University of North Carolina School of Medicine, Chapel Hill, NC

**Keywords:** Drug-Induced Liver Injury, Drug-Induced Liver Injury Network, Acute Liver Injury, Herbal and Dietary Supplements, Greater Celandine

## Abstract

Greater celandine (*Chelidonium majus*) leaf extracts have been used for centuries as a natural remedy for various gastrointestinal symptoms. Greater celandine is associated with several case reports of hepatotoxicity, mainly from Europe. No cases from the United States have been identified. We present a case of acute hepatitis from greater celandine in the United States in a 72-year-old man taking this herbal supplement for nausea. In patients presenting with acute liver injury, gastroenterologists should be aware of this herb and reminded to assess for herbal and dietary supplement hepatotoxicity, especially in those remedies used to treat common gastrointestinal symptoms.

## INTRODUCTION

Idiosyncratic drug-induced liver injury (DILI) accounts for approximately 13% of acute liver injuries (ALI).^1,2^ The incidence of herbal and dietary supplement (HDS)-related DILI has nearly tripled in the United States since 2004.^3^ Even in the absence of alternative etiologies of ALI, diagnosing DILI is challenging in patients with polypharmacy and other confounding variables.

**Figure 1.**
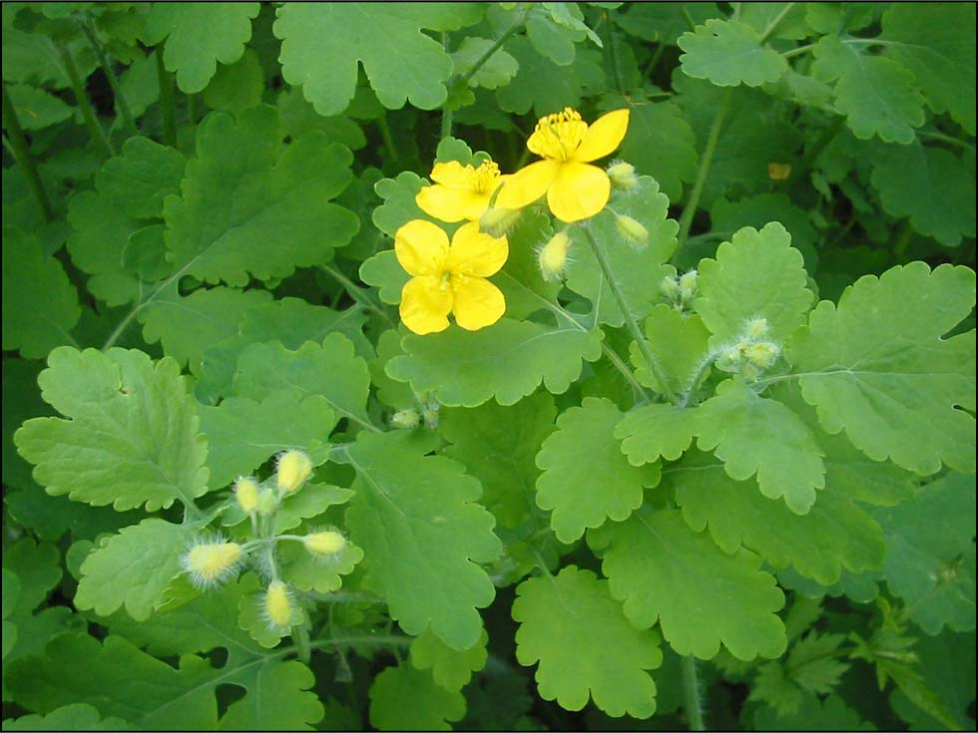
Greater celandine (*Chelidonium majus*) flower.

Greater celandine (*Chelidonium majus*) is a flowering plant in the Poppy family (Papaveraceae) indigenous to Eurasia^4^ (Figure 1). Greater celandine leaf extracts contain several biologically active alkaloids with reported antispasmodic and mildly sedating properties.^4^ Greater celandine has been used in homeopathy for centuries for gastrointestinal symptoms including nausea, dyspepsia, and biliary colic.^1,4,5^ Case reports, primarily in Europe, describe greater celandine causing idiosyncratic hepatotoxicity.^4,5^ We present to our knowledge the first reported case of DILI attributed to greater celandine in the United States

## CASE REPORT

A 72-year-old man with diabetes, diabetic neuropathy, hypertension, hyperlipidemia, irritable bowel syndrome, and chronic pain presented to the hospital with several days of malaise, dark urine, and jaundice. He had no history of liver disease, abnormal liver tests, alcohol, or substance use. His home medications included acetaminophen-hydrocodone, aspirin, atorvastatin, glipizide, insulin glargine, and metformin (Table 1). The only new prescribed medication was liraglutide, which was started 2 weeks before admission. Three months before admission, he started using several HDS for chronic abdominal pain and nausea including *Nux vomica*, *Arnica montana*, and greater celandine, 5 pellets 3 times a day (approximately 6.6 mg/d). His chronic gastrointestinal upset was attributed to irritable bowel syndrome as prior endoscopy, and abdominal imaging were normal.

**Table 1.** Patient's medications and supplements

Medication/product	Dose/frequency	LiverTox likelihood score^a^
Acetaminophen-hydrocodone	5–325 mg 2× daily PRN	A (in high doses; ≥4 g/d)
*Arnica Montana*	20 cc 4× daily	E
Aspirin	81 mg daily	A (in high doses; 1,800–3,200 mg/d)
Atorvastatin	80 mg daily	A
*Chelidonium majus*	5 pellets (30 cc; 2.21 mg) 3× daily	B
Glipizide	10 mg 2× daily	C
Insulin glargine	25 units nightly	A (in high doses; >100 units daily)
Metformin	1,000 mg 2× daily	B
*Nux vomica*	5 pellets (2.21 mg) 4× daily	E
Liraglutide	1.2 mg daily	E

^a^ Likelihood of DILI: A = well known, >50 cases including case series, characteristic signature; B = known or highly likely, 12–50 cases including small case series, characteristic signature; C = probably linked, <12 cases without case series; D = possible, <3 case reports; E = not believed or unlikely, no evidence/unconvincing single case reports; X = unknown.^17^

**Figure 2.**
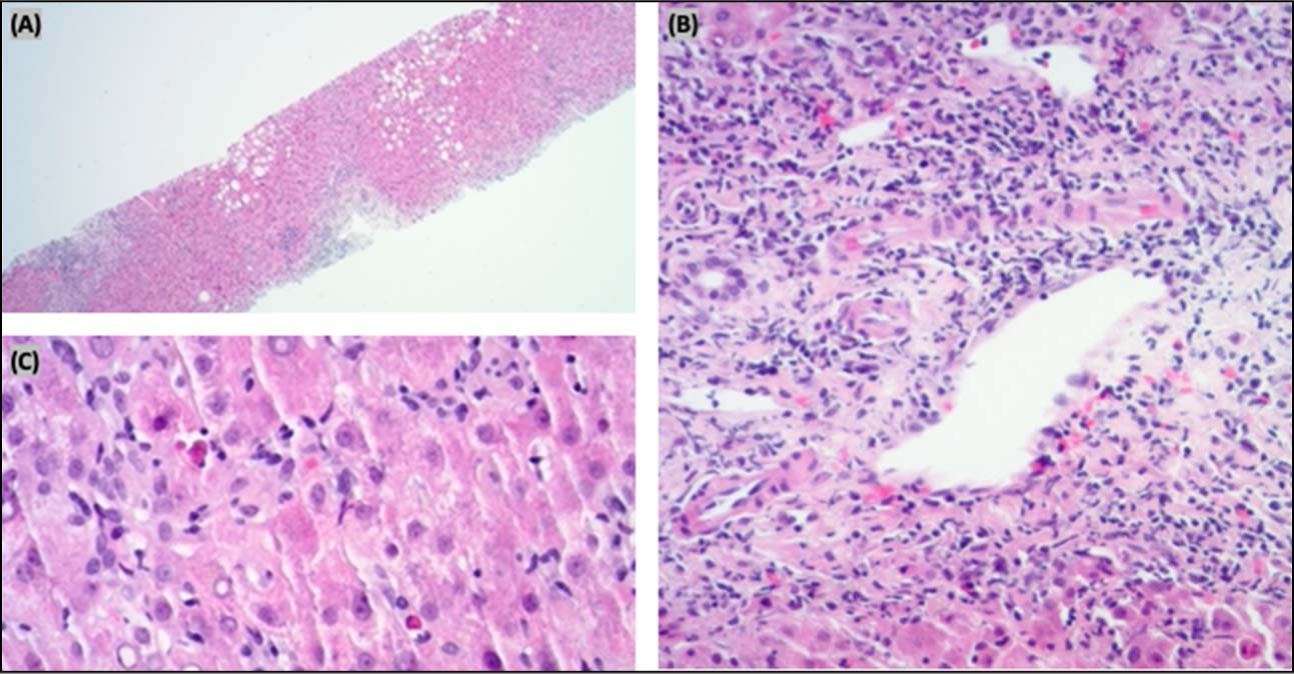
Liver biopsy. (A) Hematoxylin and eosin (H&E) stain at 2× shows intact hepatic architecture with mild fatty change and evidence of variably dense inflammation within lobules and portal tracts. (B) H&E stain at 10× shows portal tract containing lymphocytic inflammation with some eosinophils and evidence of interface hepatitis. (C) H&E stain at 20× shows lobular lymphocytic inflammation with eosinophils and hepatocyte necrosis.

On presentation, vital signs were within normal limits and physical examination was unremarkable except for jaundice. There was no evidence of hepatic encephalopathy, hepatosplenomegaly, or ascites. Serum total bilirubin was 12.3 mg/dL, alkaline phosphatase 507 U/L, alanine aminotransferase 974 U/L, and aspartate aminotransferase 1,119 U/L consistent with hepatocellular liver injury (*R* value 5.8). Broad workup including viral and autoimmune hepatitis serologies was negative (Table 2). Abdominal ultrasound and computed tomography were normal, ruling out obstruction. Liver biopsy showed hepatitis, lymphocytic inflammation with eosinophils, hepatocyte necrosis, grade 3 activity, stage 1 fibrosis, and mild fatty changes (Figure 2).

**Table 2.** Liver tests trend and serologic evaluation of acute liver injury

Liver tests and reference range	Admission (HDS stopped)	5 d (discharge)	11 d	6 wk	8 mo
Albumin (3.7–4.7 g/dL)	2.5	2.2	3.3	3.7	4.4
Alkaline phosphatase (44–121 U/L)	507	439	305	111	92
ALT (0–44 U/L)	974	549	106	12	16
AST (0–40 U/L)	1,119	312	49	12	17
Direct bilirubin (0–0.4 mg/dL)		10.7	2.85	0.69	0.28
Total bilirubin (0–1.2 mg/dL)	12.3	14.7	3.9	1.3	0.9
Total protein (6–8.5 g/dL)			7	6.7	6.7
Platelets (150–450× 10e^3^/µL)	165	211			184
INR (0.9–1.2)	1.1	1.0			1.0

Additional serologic evaluation	Value	Interpretation
Hepatitis A IgM antibody	Negative	No evidence of acute hepatitis A infection
Hepatitis B surface antigen Hepatitis B surface antibody Hepatitis B core IgM antibody Hepatitis B core total antibody	Negative Negative Negative Negative	No evidence of past or present hepatitis B infection, nonimmune
Hepatitis C antibody Hepatitis C RNA	Negative Negative	No evidence of acute or chronic hepatitis C infection
Epstein-Barr virus antibody IgG	210 U/mL	Elevated IgG level with normal IgM indicates past infection
Epstein-Barr virus antibody IgM	20.5 U/mL	
Antimitochondrial antibody	<20 units	Negative, unlikely to have autoimmune hepatitis or primary biliary cirrhosis
Antismooth muscle antibody, IgG	11 units	Normal, unlikely to have autoimmune hepatitis
IgG total serum	1,198 mg/dL	Normal circulating IgG level
IgM total serum	75 mg/dL	Normal circulating IgM level
Acetaminophen level	<3.0 μg/mL	No evidence of acute acetaminophen toxicity
Salicylate level	<5.0 μg/mL	No evidence of acute salicylate toxicity
Ceruloplasmin level	34.3 mg/dL	Mildly elevated and not consistent with Wilson disease
Alpha-1-antitrypsin	177 mg/dL	Normal, no evidence of alpha-1-antitrypsin deficiency
Ferritin	1,169 ng/mL	Elevated, but liver biopsy was negative for increased parenchymal iron making hemochromatosis unlikely. This is also an acute phase reactant
Transferrin saturation	53.6%	Mildly elevated, but liver biopsy was negative for increased parenchymal iron making hemochromatosis unlikely

ALT, alanine aminotransferase; AST, aspartate aminotransferase. INR, international normalized ratio; IgM, immunogloblulin M; IgG, immunoglobulin G

**Figure 3.**
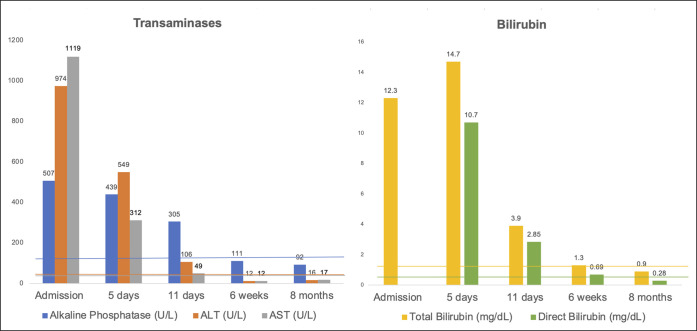
Transaminase and bilirubin trend.

DILI was the suspected etiology of transaminase elevation, and all HDS products were discontinued, none were restarted. Liver tests improved, and he was discharged. Within 2 months of hospitalization, his liver tests normalized and remained normal at the 8-month follow-up (Figure 3).

## DISCUSSION

HDS are widely used yet often overlooked and underreported. The incidence of HDS-related DILI in the United States almost tripled from 7% (2004) to 19% (2012), and HDS sales in the United States increased by 17.3% in 2020.^3,6^ A prospective study by the Drug-Induced Liver Injury Network found 15.5% of DILI were caused by HDS.^7^ As HDS use increases, providers must be aware of commonly used herbs and potential toxicities.

Greater celandine is available in the United States and advertised as a safe and effective antiemetic for adults and children.^8^ The leaf extracts of greater celandine contain over 20 biologically active isoquinoline alkaloids including benzophenanthridines, protoberberines, and hydroxycinnamic acid derivatives, although the exact chemical(s) responsible for hepatotoxicity is unknown.^4,5,9^ Through an unclear mechanism, these alkaloids have antispasmodic and mildly sedating properties and have been shown in animal studies to increase bile flow.^4,5,9^ Greater celandine is used for many gastrointestinal symptoms such as nausea, dyspepsia, and gallbladder disease.^4,5^ Alternative uses include asthma, bronchitis, skin conditions, and weight loss.^4^

Greater celandine is a known hepatotoxin (likelihood score B) with many idiosyncratic features described in more than 40 case reports.^4,5^ As greater celandine is native to Eurasia, nearly all cases in the medical literature are from Europe, no cases have been described in the United States^4,5^ Dosing associated with greater celandine DILI is difficult to determine as HDS are not strictly regulated, although a clinical review of 43 cases found the average daily dose was 10 mg.^5^ Clinically greater celandine associated DILI resembles acute hepatitis with jaundice and severely elevated transaminases in a hepatocellular pattern.^4,5^ Liver histology typically shows hepatitis and necrosis, the stage 1 fibrosis in this case was likely from prevalent hepatic steatosis.^5^ Hepatotoxicity occurs within 1–6 months of taking the herb and rapidly resolves without intervention after the product is discontinued.^4,5^ Autoantibodies are not typically present but can be in low levels, although clinically greater celandine DILI does not resemble autoimmune hepatitis or require corticosteroids.^4^ This patient started using greater celandine approximately 6.6 mg/d 3 months before ALI and his clinical course, and liver tests and biopsy resembles other described cases of greater celandine DILI from Europe.^4,5^

Still, establishing causality for DILI in this case was challenging due to many confounding factors. He recently started liraglutide, was taking known hepatotoxic medications, and used several HDS (Table 1). Despite the temporal association of liraglutide initiation and liver injury, DILI from glucagon-like peptide 1 agonists is rare (likelihood score E).^10–13^ In large clinical trials, there was no significant difference in transaminase elevation between liraglutide and placebo.^10^ Over the last decade, only single case reports of glucagon-like peptide 1 agonist hepatotoxicity exist.^10–13^ One case reported liraglutide-associated autoimmune hepatitis that did not resolve with discontinuation of liraglutide and required long-term corticosteroids.^13^ Otherwise, there are 2 published cases of liraglutide-associated DILI that demonstrated hepatocellular injury and resolved after discontinuing the drug.^11,12^ The home hepatotoxic medications with likelihood score A must be taken in high doses to cause DILI, and he was not taking such doses (Table 1). Metformin (likelihood B) can causes minor transaminase elevation but rarely causes clinically apparent ALI.^14^ Importantly, he tolerated all home medications for years with no history of elevated liver tests or dose changes, and these medications were continued as liver tests improved, rendering these agents as unlikely causes of his ALI. *Nux vomica* and *Arnica montana* have not been implicated in causing liver injury.^15,16^ Therefore, the most likely product responsible for this patient's ALI is greater celandine. This case was enrolled in the Drug-Induced Liver Injury Network and adjudicated by the Causality Committee to be highly likely DILI from greater celandine with a Roussel Uclaf Causality Assessment Method score of 9 indicating greater celandine DILI is highly probable.

This case describes greater celandine associated hepatotoxicity with idiosyncratic features consistent with other described cases in Europe. Also highlighted are the challenges of determining causality for DILI and consequently the importance of thorough medication history including specific HDS and homeopathic remedies. Gastroenterologists should be aware that greater celandine is available in the United States and used for common gastrointestinal symptoms to provide counseling on the potential risks of this herb.

## DISCLOSURES

Author contributions: S. Power: writing and editing. AS Barritt: writing editing and supervision. A. Sidney Barritt IV is the article guarantor.

Financial disclosure: The Drug Induced Livery Injury Network is funded by the National Institutes of Health.

Previous presentation: This case was presented as a poster at the October 22nd, 2023 American College of Gastroenterology Annual Meeting; Vancouver, BC, Canada.

Informed consent was obtained for this case report.
